# Residential energy expenditures and the relevance of financial inclusion across location, wealth quintiles and household structures

**DOI:** 10.1016/j.heliyon.2024.e26710

**Published:** 2024-02-23

**Authors:** Martinson Ankrah Twumasi, Frank Adusah-Poku, Alex O. Acheampong, Eric Evans Osei Opoku

**Affiliations:** a College of Economics, Sichuan Agricultural University, Chengdu, Wenjiang, China; b Department of Economics, Kwame Nkrumah University of Science and Technology, Kumasi, Ghana; c Environment and Natural Resource Research Initiative (ENRRI-EfD Ghana), Accra, Ghana; d Bond Business School, Bond University, Gold Coast, Australia; e Centre for Data Analytics, Bond University, Gold Coast, Australia; f Nottingham University Business School China, University of Nottingham, Ningbo, China

**Keywords:** Energy expenditure, Financial inclusion, Rural, Ghana

## Abstract

This paper examines the relative role of financial inclusion in enhancing households’ ability to spend on energy consumption across rural and urban locations. It uses comprehensive household data from Ghana and employs the ordinary least square (OLS) as well as an instrumental variable estimation technique. Endogeneity of financial inclusion is instrumented using distance to the nearest bank. Our findings suggest that a standard deviation increase in financial inclusion contributes to an improvement in residential energy expenditure by 1.2835 standard deviations. This finding is robust to different methods for resolving endogeneity and alternative weighting schemes in the financial inclusion construct. Among the different sources of energy for lighting and cooking, financial inclusion increases expenditure on LPG and electricity more than the others. Financial inclusion increases the ability to spend more on residential energy in urban, poorest, and female-headed dual-parent households. Household net income is a key pathway through which financial inclusion affects residential energy expenditure.

## 1 Introduction

This study aims to investigate the impact of financial inclusion on the residential energy expenditure of households in Ghana, a country in sub-Saharan Africa (SSA). Energy for cooking and lighting is essential to every household in SSA and the rest of the world. Despite the numerous attempts to transition households to cleaner and modern cooking fuels in SSA and other developing regions across the world, there continues to be a large dependence on traditional cooking fuels like charcoal and firewood. For instance, the percentage of people using clean cooking fuels in SSA only increased from about 9% in 2000 to nearly 19% in 2021 [1]. In South Asia, nearly 63% of the population has had access in 2021. In countries like Senegal, Sri Lanka, Lesotho, Philippines, and Pakistan, only about 29%, 32%, 41%, 48%, and 50.7%, respectively, of the population had access to clean cooking fuels in 2021 [2]. In Ghana, where this study is conducted, the share of households that depend on traditional cooking fuels declined by less than 5 percentage points from 89.8% in 2006 to 84.9% in 2013 [3]. In 2021, only about 30% of the population had access to clean cooking fuels and technologies [2]. Also, access to electricity continues to be limited in many countries within the SSA region and other developing countries. Data show that over 600 million people do not have electricity access, with 7 million located in Ghana [4]. In Madagascar, Mauritania, Rwanda and Mali just about 35%, 48%, 49% and 53%, respectively, of the population had access to electricity in the year 2021 [2]. The implication is that a chunk of the population relies on traditional energy sources. The heavy dependence on traditional cooking fuels is associated with exposure to different health-related matters [5,6]. On the other hand, clean cooking fuels have been found to save lives, improve livelihoods, empower women, reduce indoor air pollution, reduce time spent on cooking, increase the productivity of the poor and protect the environment [[7], [8], [9], [10]]. The high dependence on traditional or dirty cooking fuels, along with the lower use of electricity, is mainly due to the expensive nature of these fuels [11].

In the literature, some studies have provided reasons for the less patronage of clean cooking energy, such as electricity, Liquefied Petroleum Gas (LPG) and the overreliance on dirty cooking energy, such as firewood, charcoal, and kerosene. For instance, Karimu, Mensah [11] indicated that LPG's price limits households' usage of LPG. A similar result was found by Adusah-Poku and Takeuchi [12]. On the contrary, household income increases the likelihood of modern cooking fuel usage while decreasing the adoption of traditional cooking fuels in Ghana [11,[13], [14], [15], [16]]. Another important factor that limits clean energy use or increases energy expenditure is energy price. For instance, Foster, Tre [17], using data from Guatemala, indicated that increasing energy prices is associated with less access to electricity and increases fuel poverty. In another study, Mensah and Adu [15] indicated that energy price affects the choice of energy usage. The authors showed that the price of LPG, fuelwood and charcoal reduces firewood usage; however, the price of LPG increases charcoal, LPG usage and other solid fuel usage. Also, using data from China, Hang and Tu [18] showed that the price elasticity of coal, oil and aggregate energy were negative, signifying that the relative different energy prices lead to a decrease in coal, oil and aggregate energy intensities. As high energy costs and prices deter household usage of modern cooking fuels, the question that motivates this study is can financial inclusion, defined as easy access to financial services for households and businesses, improve household spending on clean cooking energy while reducing dirty cooking fuels? In relation to this question, some emerging studies have analysed the role of financial inclusion on household energy poverty or access to electricity and clean cooking fuels and energy [[19], [20], [21], [22]]. However, among these studies, the impact of financial inclusion on residential energy expenditure has received less attention. This study, therefore, addresses this research gap by using a nationally representative household survey dataset to assess the impact of financial inclusion on residential energy expenditures in Ghana.

This study is motivated to focus on Ghana because residential energy consumption in this country has been increasing over the past decade. For instance, there was an increase in residential electricity consumption in Ghana from 1996 GWh in 2007–3932 GWh in 2016 [23]. The increase has been attributed to several factors, such as population, urbanization, appliance ownership and technological advancement [24,25]. By implication, residential energy expenditures are expected to follow an upward trend as residential electricity consumption is a key component of residential energy expenditure. Although electricity use has enormous benefits, monthly electricity bills and connection costs can hinder realizing these benefits. This explains why some households lack access to electricity even though their communities are connected to the national grid [12]. This tends to raise issues about households’ purchasing power and their ability to pay for increases in residential energy expenditure. It even becomes a bigger problem if households devote a larger proportion of their income to residential energy demand, forcing them to forgo other consumption activities. Furthermore, the importance of male-female household headship structure and rural-urban dynamics are being increasingly recognized in the household wealth and poverty narrative, especially in Africa and other developing countries where men and urban residents mostly have an advantage in the ownership of and access to resources, including assets, loans, and bank accounts [[26], [27], [28]].

Also, financial inclusion in Ghana has seen significant progress, although access to financial services is limited to a significant section of the populace. Access to formal financial services increased from 41% in 2010 to 58% in 2015, increasing by 17 percentage points [27]. Banks provide most of these financial services, although mobile money services have seen a rapid increase, particularly post-2010. The heterogeneity of access to these financial services can be observed geographically across the country. For instance, residents in the five poorest regions of Ghana and those living in rural areas remain the most financially excluded group [27].^1^ The type of financial service used also differs from one location to the other. Rural and poor residents use more informal financial services than urban and nonpoor [27]. In the quest to increase the availability of affordable and quality financial services to reduce economic vulnerability, the government of Ghana, particularly the Bank of Ghana, have embarked on a series of policy reforms over the years. Paramount among these reforms is the introduction of the 2018–2023 National Financial Inclusion and Development Strategy (NFIDS), which is aimed at addressing the barriers that prevent people from accessing financial products and services.^2^

Our research is unique and contributes to the literature in the following ways: First, this study contributes to the energy poverty literature by examining the impact of financial inclusion on total energy expenditure using two rounds of nationally representative household survey data from Ghana. In addition to total energy expenditure, this study extends the analysis by considering the impact of financial inclusion on household expenditure on different energy such as firewood/charcoal, LPG, electricity and kerosene. Second, while financial inclusion and energy expenditure differ between rural and urban locations and poor and wealthy households, this study also provides insight into the role of financial inclusion on energy expenditure between rural and urban households and poor and wealthy households. Third, this study extends the literature by analyzing the factors mediating financial inclusion's impact on households' energy expenditure. Finally, this study deploys a battery of robust econometric techniques to unravel the implication of financial inclusion for households' energy expenditure. Specifically, our study applies instrumental variable (IV) techniques to address endogeneity. In addressing endogeneity, the study uses distance to the nearest bank as an external instrument to account for the exogenous impact of financial inclusion on energy expenditure. We further test the robustness of results by assigning different weights to the variables used to generate financial inclusion. Overall, this study is policy-relevant as its outcomes would be relevant for alleviating energy poverty in Ghana.

This study's findings indicate that financial inclusion significantly and positively impacts residential energy expenditure. The results also indicate that financial inclusion increases residential energy expenditure more for urban households than their rural counterparts. At the same time, financial inclusion enhances poor households' residential energy expenditure more than wealthy households. Our ﬁndings further revealed that households' net income is an important channel through which financial inclusion transmits to residential energy expenditure. These findings are robust to sensitivity checks, including alternative weighting schemes for the financial inclusion index.

The remaining sections of the paper are organized as follows: Section 2 explains the conceptual link between financial inclusion, energy use, and residential energy expenditure. Section 3 describes the methodology. Section 4 presents and discusses the empirical results. We conclude in Section 5.

## 2 Review of the conceptual and empirical literature

This section reviews some empirical and theoretical studies on the subject, focusing more on the channels which mediate the financial inclusion-residential energy expenditure linkage. Financial inclusion is defined as all individuals and businesses having access to a range of financial products and satisfying their needs in a reasonable, suitable, reliable, and sustainable manner [29]. These channels are the main sources of income and entrepreneurial activities. As such, the review is grouped into four main subsections. The first section presents the general findings of prior studies on the link between financial inclusion and energy poverty.^3^ The next two subsections discuss income and entrepreneurship as potential channels, while the fourth focuses on other potential mechanisms.

### 2.1 Conceptual relationship between financial inclusion and residential energy expenditure

The ability to pay for residential energy expenditure can be influenced by financial inclusion, particularly through economic growth. Financial inclusion enhances economic growth by first promoting financial sector development. According to Bayar, Ozkaya [30], there are direct and indirect channels through which financial sector development can influence energy use. The direct channel is through its effect on economic growth. Another channel is using funds the financial sector provides to purchase energy products, thereby increasing energy use and residential energy expenditures. Also, a robust financial sector can assist economic units such as firms and households to hedge during periods of energy price fluctuations. Although the financial sector development anchored by financial inclusion increases energy use, the opposite could also occur. Financial sector development can assist in reducing energy use through the provision of funds to firms to produce energy-efficient technologies and products [30]. The expansion of the financial sector positively affects investments, savings, and economic growth [31]. Expanding the economy via the positive effects of financial inclusion on savings and investments improves income levels [32]. The higher income levels increase the demand for goods and services, including energy services, possibly increasing the ability to spend on residential energy expenditures. Unfortunately, the literature on the effect of financial inclusion on residential energy expenditure is non-existent. However, it is inferred from the aforementioned that financial inclusion could increase residential energy expenditures.

Also, financial inclusion could influence residential energy expenditure through entrepreneurship. Entrepreneurship has long been an important engine of inclusive development [33]. For most developing countries where youth unemployment and joblessness constitute a major socio-economic problem, policymakers have been encouraged to embrace financial inclusion to enhance entrepreneurship development to solve some of these socio-economic problems [34]. The link between financial inclusion and entrepreneurship cannot be overemphasized. This is because the lack of finance is one of the key factors that constrain entrepreneurial activities, particularly for low-income groups. Several empirical and theoretical studies reveal the significant contribution of financial inclusion in entrepreneurial activities. Financial inclusion ensures that all economic agents have the same opportunity [35]. Financial inclusion can create value for small and medium businesses, potentially reducing poverty and inequality [36,37]. Financial inclusion has also been found to reduce the cost of starting businesses, especially businesses that cannot access external funds or self-finance their businesses [38]. Financial inclusion allows firms to make innovations and expand their businesses [[39], [40], [41]].

Theoretically, financial inclusion development has the potential to mitigate credit constraints faced by entrepreneurs by decreasing information failure that occurs in financial transactions [42]. Although some studies did not identify the channels through which financial inclusion can improve entrepreneurial activities, most confirm a direct association between financial inclusion and entrepreneurship [34,[43], [44], [45], [46]]. Other empirical studies also decompose entrepreneurship into segments such as women's entrepreneurship and youth's entrepreneurship and attempt to ascertain the role of financial inclusion in promoting the activities of these segments [44,[47], [48], [49], [50]].

Further, financial inclusion can affect residential energy expenditure by improving education, health, and labour outcomes and reducing poverty. Some empirical studies have been conducted to ascertain financial inclusion's role in reducing poverty [21,28,[51], [52], [53], [54]]. The conclusion of these studies, albeit with different methodological approaches, concludes that poverty can be reduced via financial inclusion. However, most of these studies do not proceed to ascertain the possible channels through which financial inclusion influences poverty. However, some possible channels are identified but not rigorously and econometrically tested. For instance, Burgess and Pande [51] attributed the poverty reduction of financial inclusion to increased bank branches in rural areas. Churchill and Marisetty [54] also identified access to insurance as the strongest effect of financial inclusion on poverty. Very few studies attempt to identify some possible channels. For instance, Koomson and Danquah [21] identified the potential channels to be consumption poverty and household net income; Li [52] identified the possible channel to be relative income (income comparisons with peers). Poverty is a major constraint to the uptake of modern energy sources and their expenditures.

**Fig. 1:**
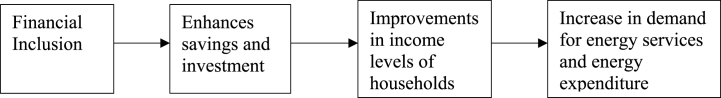
A simple conceptual framework.

Source: Authors' Construct

Other inquiries have also been made on the role played by financial inclusion in improving education, health, and labour outcomes. These studies include Ajefu, Demir [55], Koomson and Ibrahim [56], Gyasi, Adam [57], Matekenya, Moyo [58], and Stein and Yannelis [59]. The general conclusion from these studies is that financial inclusion facilitates investment in health, thereby improving various health outcomes. For instance, Ajefu, Demir [55] examined the financial inclusion effect on mental health in Nigeria and identified the possible channels mediating the financial inclusion-mental health linkage. The results show that financial inclusion positively influences mental health by passing through channels such as food expenditure and remittances. Similar results were found by Gyasi, Adam [57], concluding that financial inclusion improves various health outcomes such as psychological distress and self-related health. It has been found that financial inclusion encourages households to save towards education and that financially included households have a higher probability of sending their children to school [59]. With regard to labour outcomes, it has also been found that financial inclusion encourages households to access funds that can be used to establish household enterprises [56]. Few studies have been conducted on the financial inclusion-energy poverty linkage (measured through deprivation). The results so far show that financial inclusion reduces energy poverty and allows greater energy efficiency usage [20,21,60,61]. For instance, a study by Dogan, Madaleno [20] in Turkey found that financial inclusion reduces energy poverty through channels such as health and income compared to channels such as consumption and income poverty, as found by Koomson and Danquah [21]. This reduction effect suggests a rise in the uptake of modern energy sources and the expenditure on these energy sources. Fig. 1 presents a simple conceptual framework to explain how financial inclusion has the potential to improve residential energy expenditure. This is based on the potential channels explained in this section.

### 2.2 Review of empirical studies on financial inclusion and energy poverty

Energy poverty has been a major development topic, particularly after the 1990s. There is a consensus among policymakers globally that energy poverty should be reduced to the barest minimum. This has ignited interest among researchers in finding ways to achieve this goal. More recently, researchers have been focusing on how financial inclusion can reduce the incidence of energy poverty, both at the micro and macro levels. This is because several studies have confirmed the positive association between incomes and the adoption and use of modern energy services, which help reduce energy poverty [11,[13], [14], [15], [16]].

At the macro level, Zhao, Sun [62] used time series data from 1990 to 2021 to examine the effect of financial inclusion on energy poverty in the Republic of Korea. Their empirical results showed that financial inclusion is key to reducing the incidence of energy poverty both in the short and long run. Similar results have been found in more recent studies by Ref. [63] and Said and Acheampong [22] using panel data from South Asian Economies and panel data from twenty-three sub-Saharan African countries, respectively.

Other studies have also tested the effect of outcomes of financial inclusion policies on energy poverty. For instance, Dong, Taghizadeh-Hesary [64] found that inclusive financial development, an outcome of financial inclusion policies, is key to eradicating energy poverty. Their study applied the differential generalized method of moments on data from thirty Chinese provinces from 2004 to 2017. Other studies, such as Khan and Majeed [65], have also collaborated on this result, which found financial sector development to significantly reduce energy poverty using a sample of 110 developing countries. Generally, macro studies on the effect of financial inclusion and its outcome indicators on energy poverty have shown that countries can pursue financial inclusion policies and programs to eradicate energy poverty. The major limitation of these macro studies is the failure to explain and test the possible transmission channels through which financial inclusion helps reduce energy poverty.

The findings from the micro studies are not different from the macro studies. Dogan, Madaleno [20] relied on Turkish household data in 2018 and concluded that financial inclusion significantly reduces the incidence of energy poverty in Turkey. Similar results were found by Koomson and Danquah [21] in Ghana and Sen, Karmaker [66] in Bangladesh. However, we do not find studies on the link between financial inclusion and residential energy expenditures, which makes our study unique.

## 3 Data and variables

The study uses data extracted from the sixth and seventh rounds of the Ghana Living Standards Survey (GLSS6 and GLSS7), which were respectively conducted in 2012/13 and 2016/17 by the Ghana Statistical Service (GSS) (Ghana Statistical Service [GSS] et al., 2019; GSS, 2014). Using a two-stage probability sampling approach, both studies covered modules on demographic characteristics, energy and fuel consumption, health, agriculture, employment and time use, financial and durable assets, access to resources, and others (see GSS, 2014; 2019 for all modules covered). Across all the then 10 regions of Ghana, the GLSS6 and GLSS7, respectively, covered 18,000 and 15,000 households in 1200 and 1000 enumeration areas (clusters). In respective terms, 93.2% and 93.4% of respondents completed the GLSS6 and GLSS7 surveys, which resulted in final household sample sizes of 16,772 (GLSS6) and 14,009 (GLSS7).

Combining the sections containing our variables of interest in the two surveys, we obtained a reduced sample of 30,597 households—16,751 (GLSS6) and 13,846 (GLSS7). We use a pooled cross sectional dataset because the households in GLSS6 and GLSS7 are different. Due to missing data, estimations with the most observations had pooled households of 23,678—GLSS6 (16,742) and GLSS7 (6936). The large reduction in observations after the regression analysis is explained by the financial inclusion variable in the GLSS7, which has 6910 missing data owing to non-responses associated with the indicators that made up the index. Summary statistics of the variables used are reported in Table A1 Appendix A1.

From the Appendix Table A1, the mean value for total energy expenditure is 306.183 Ghana cedis. Examining the expenditure for different components of energy, it is obvious that households spend more on electricity, followed by firewood/charcoal, Gas and Kerosine. Thus, the average household expenditure for electricity is 182.798 Ghana cedis, firewood/charcoal is 92.956 Ghana cedis, Gas is 24.382 Ghana cedis, and Kerosine is 6.055 Ghana cedis. With regards to financial inclusion, 35.04% of household heads are financially included, while 64.96% of households’ heads are financially excluded.

Also, the summary statistics show that approximately 29.12% of household heads were females, while 70.88% of the respondents were males. At the same time, 57.84% of the households' heads were married, and 48.40% of the households' heads were educated. The mean age for the household head is 46 years, and the average household size is 4. Based on the statistics, 56.09% of the household heads responding to the survey questions were found in rural areas. Regarding employment status, 8.89% of household heads were on retirement/inactive, 21.8% were employees, and 64.71% were self-employed. Also, about asset variables, 28.3% of the household heads own a fridge/freezer, and 66.51% own a radio or television. Regarding the nature of the house, 9.77% of the household heads lived in semi-detached/flat/apartments, 55.55% lived in compound houses, and 16.77% lived in Huts/Buildings/others. The average number of rooms in a house is 2. Also, the average distance to the nearest bank is 14.33 km. Finally, the average household head's net income is 2162.777 Ghana cedis.

### 3.1 Financial inclusion

Financial inclusion is measured using the World Bank's multidimensional concept of financial inclusion, which is similar to that used in past studies [21,67,68]. Financial inclusion measurement is in four dimensions in this paper: bank or mobile money account ownership, ownership of insurance, access to credit/loans, and receiving financial remittances via bank or mobile money. A generated financial inclusion score is attained using Eq. (1), assigning an equal weight of 0.25 to each dimension. A unit increase in a household's financial inclusion score reflects an increase in its level of financial inclusion. Following previous studies, we employ a cut-off of 0.5 to ascribe a value of 1 to a household with a financial inclusion score of more than 0.5 and 0 otherwise.

(1) $$d_{i}=w_{1}I_{1}+w_{2}I_{2}+\cdots +w_{n}I_{n}$$

where $d_{i}$ is the household financial inclusion score $I_{i}=1$ if a household provides an affirmative response for indicator i, and $I_{i}=0$ if otherwise. $w_{i}$ is the weight attached to indicator i with $\sum_{i=1}^{d}w_{i}=1$.

### 3.2 Residential energy expenditure

Residential energy expenditure is measured as the total value of household expenditure on different energy sources used for lighting and cooking. These include expenditures on firewood, charcoal and other fuels, kerosene, LPG, and electricity. Apart from the total residential energy expenditure, we also decompose the expenditures into their main components for analysis. Considering the large dispersions in the total residential energy expenditures across different income groups and locations, we use the logged version of the residential energy expenditures in the regression analysis to obtain unbiased estimates (see means and standard deviations in Table A1).

## 4 Empirical specification and methods

To estimate the relationship between financial inclusion and residential energy expenditure we use the baseline model specified in Eq. (2).

(2) $${REE}_{i}=\beta_{1}{FI}_{i}+\sum_{n}\beta_{n}X_{n,i}+\mu_{r}+\varepsilon_{i}$$

where REE is residential energy expenditure for households i, and FI represents the financial inclusion status of a household. X is a set of control variables identified as determinants of residential energy expenditure (Adusah-Poku & Takeuchi, 2019c; Eakins, 2013; Longhi, 2015). $\mu_{r}$ is a regional fixed effect, and ε is a random error term. For the baseline analysis, we use the Ordinary Least Squares (OLS) method. The main advantage of the OLS method is that it is the best linear unbiased estimator. However, the major weakness of the OLS is that it assumes strict exogeneity of the variables; hence, any plausible endogeneity makes its estimates biased.

### 4.1 Potential endogeneity

The literature exploring the effect of financial inclusion on welfare and other outcome variables points to the endogeneity problem associated with financial inclusion [28,67]. We suspect bi-causality as being the source of the endogeneity. On one side, the financial resources associated with financial inclusion can increase households’ ability to pay for energy expenditure. On the other side, household heads who want a steady energy supply for lighting and cooking may be motivated to become financially included by saving for it or obtaining a credit/loan from a bank. Others may have obtained various forms of financial accounts or engaged with the financial system to receive remittances, which can be used to cater for household expenses on energy.

As applied in previous studies, we address the potential endogeneity problem by using an instrumental variable (IV) estimation procedure that employs distance to the nearest bank as an instrument [28,54,67]. Based on the validity of the instrument, evidence shows that distance to the bank has a direct inverse relationship with financial inclusion [21,28,67,69]. Conversely, the distance it takes to reach a bank is not expected to directly influence residential energy expenditure unless it occurs through financial inclusion. The IV estimation procedure follows a two-stage procedure. In the first stage, the financial inclusion status of the household is determined by a set of exogenous covariates and our instrument is defined as follows:

(3) $${FI}_{i}=\alpha_{1}D_{i}+\sum_{n}\alpha_{n}X_{n,i}+\mu_{r}+\varphi_{i}$$

Where $D_{i}$ is the instrument (distance to the bank), $\alpha_{1}$ and $\alpha_{n}$ are parameters to be estimated and $\varphi_{i}$ is the random error term. In the second stage, residential energy expenditure is regressed on the predicted values of financial inclusion obtained from estimating Eq. (3) and the set of exogenous covariates specified in Eq. (4) as:

(4) $${REE}_{i}=\gamma_{1}{\hat{FI}}_{i}+\sum_{n}\gamma_{n}X_{n,i}+\mu_{r}+\zeta_{i}$$

Where ${\hat{FI}}_{i}$ is the predicted values of financial inclusion from Eq. (3), $\gamma_{1}$ and $\gamma_{n}$ are parameters to be estimated and $\varsigma_{i}$ is the random error term.

Apart from using instrumental variable estimation to resolve endogeneity, we test for the robustness of our standard IV results by using Lewbel [70] two-stage least squares (2SLS). The Lewbel 2SLS method creates an internal instrument using heteroskedasticity in the data. In practice, one can use only the internally generated or combine external and internally generated instruments. The methods used in prior research work on financial inclusion and poverty alleviation analyses, as well as studies with weak IV, align with this approach [21,54,71].

## 5 Results

As a standard practice, we estimate baseline results using OLS. The estimated results for financial inclusion and residential energy expenditure linkage (baseline) are shown in Table 1. Results produced from the pooled GLSS6 and GLSS7 datasets are presented in Panels A, B and C, respectively. Results for total residential energy expenditure are reported in column 1, while estimates for the various components of residential energy expenditure are displayed in columns 2 to 5. In all tables, we report both standardised (in square brackets) and non-standardised results, but our interpretations are based on standardised ones to easily compare the results across models. In Column 1 of Panel A, there is an improvement in household total residential energy expenditure standard deviation (0.0700) as the standard deviation of financial inclusion rises by one. Among the different components of residential energy expenditure, a standard deviation increase in financial inclusion is associated with increases of 0.0566, 0.0832, and 0.0429 standard deviations in household expenses on firewood/charcoal, LPG, and electricity, respectively. Moreover, an improvement in financial inclusion is associated with a reduction in expenditure on kerosene.

**Table 1:** Financial inclusion and residential energy expenditure (Baseline results).

Variables	(1)	(2)	(3)	(4)	(5)
	Total Energy expenditure	Firewood/Charcoal	LPG	Electricity	Kerosene
***Panel A: Pooled data***					
Financial inclusion	0.3765***	0.2961***	0.2994***	0.2377***	−0.0350**
	(0.0283)	(0.0346)	(0.0227)	(0.0306)	(0.0154)
	**[0.0700]**	**[0.0566]**	**[0.0832]**	**[0.0429]**	**[-0.0147]**
Household head characteristics	Yes	Yes	Yes	Yes	Yes
Household variables	Yes	Yes	Yes	Yes	Yes
Year fixed effects	Yes	Yes	Yes	Yes	Yes
Regional fixed effects	Yes	Yes	Yes	Yes	Yes
Observations	23,678	23,678	23,678	23,678	23,678
R-squared	0.4031	0.1646	0.2982	0.3949	0.1092
***Panel B: GLSS6***					
Financial inclusion	0.3217***	0.2173***	0.3459***	0.2046***	**−0.0033**
	(0.0326)	(0.0405)	(0.0259)	(0.0328)	(0.0212)
	**[0.0618]**	**[0.0433]**	**[0.1002]**	**[0.0391]**	**[-0.0012]**
Household head characteristics	Yes	Yes	Yes	Yes	Yes
Household variables	Yes	Yes	Yes	Yes	Yes
Year fixed effects	No	No	No	No	No
Regional fixed effects	Yes	Yes	Yes	Yes	Yes
Observations	16,742	16,742	16,742	16,742	16,742
R-squared	0.4474	0.1626	0.3112	0.4811	0.1066
***Panel C: GLSS7***					
Financial inclusion	0.4088***	0.3949***	0.1710***	0.1527***	**−0.0308**
	(0.0544)	(0.0622)	(0.0462)	(0.0654)	(0.0154)
	**[0.0735]**	**[0.0433]**	**[0.0434]**	**[0.0250]**	**[-0.0147]**
Household head characteristics	Yes	Yes	Yes	Yes	Yes
Household variables	Yes	Yes	Yes	Yes	Yes
Year fixed effects	No	No	No	No	No
Regional fixed effects	Yes	Yes	Yes	Yes	Yes
Observations	6936	6936	6936	6936	6936
R-squared	0.3357	0.1658	0.2869	0.2841	0.0531

Robust standard errors in parentheses ***p < 0.01, **p < 0.05, *p < 0.1.

Standardised coefficients in square brackets.

In Panel B, a rise in the financial inclusion's standard deviation by one is connected to a 0.0618 standard deviation increase in household total residential energy expenditure. From Columns 2 to 5, a rise in the financial inclusion's standard deviation is linked to improvements of 0.0433, 0.1002 and 0.0391 standard deviations in household expenses on firewood/charcoal, LPG, and electricity, respectively. In Panel C (Column 1), a rise in the financial inclusion's standard deviation by one is associated with an increase in total residential energy expenditure by 0.0735 standard deviation. Columns 2 to 5 show that as financial inclusion's standard deviations increase by one, the standard deviations in household expenses on firewood/charcoal, LPG, and electricity increase by 0.0705, 0.0434 and 0.0250, respectively. The estimated findings are consistent with prior literature [16,21]. For example, Twumasi, Jiang [16] revealed that credit accessibility influences rural households' clean cooking energy consumption. The positive association between financial inclusion and residential energy expenditure is profound. The inverse relationship between financial inclusion and expenditure on kerosene exemplifies an energy transition/substitution effect where the ability to pay shifts expenditure toward cleaner energy sources.

However, the baseline results, which are based on OLS estimation, might be inconsistent or biased due to the issue of endogeneity, suggesting that applying a suitable model that can deal with the endogeneity issue is relevant. Therefore, the study applies the standard 2SLS method with distance to the nearest bank serving as an instrument and reports the results in Table 2. As expected, the first stage results imply that residents living far from the nearest bank have lower chances of being financially included (Columns 1 to 5). The F-statistics of all first-stage models are not less than the threshold of 10, depicting that the selected instrument has a strong relationship with financial inclusion (Stock & Yogo, 2002). None of these models showed an insignificant financial inclusion estimate. In addition, the standard 2SLS results are bigger than the results obtained from the baseline, suggesting that the endogeneity problem of financial inclusion results in a downward bias in the baseline results.

**Table 2:** Financial inclusion and residential energy expenditure (2SLS regression).

Variables	(1)	(2)	(3)	(4)	(5)
	Total Energy expenditure	Firewood/Charcoal	LPG	Electricity	Kerosene
***Panel A: Pooled data***					
Financial inclusion	6.9011***	1.5859*	4.8797***	6.1429***	3.6143***
	(1.2637)	(0.8498)	(0.8409)	(1.1666)	(0.6819)
	**[1.2835]**	**[0.3029]**	**[1.3566]**	**[1.1075]**	**[1.5158]**
Household head characteristics	Yes	Yes	Yes	Yes	Yes
Household variables	Yes	Yes	Yes	Yes	Yes
Year fixed effects	Yes	Yes	Yes	Yes	Yes
Regional fixed effects	Yes	Yes	Yes	Yes	Yes
**First-Stage**					
Distance to the nearest bank (km)	−0.0029***	−0.0029***	−0.0029***	−0.0029***	−0.0029***
	(0.0005)	(0.0005)	(0.0005)	(0.0005)	(0.0005)
F-statistic of first regression	41.96	41.96	41.96	41.96	41.96
Observations	23,678	23,678	23,678	23,678	23,678
***Panel B: GLSS6***					
Financial inclusion	10.8210***	3.8409**	6.7753***	6.9181***	6.8304***
	(3.2974)	(1.8183)	(2.0371)	(2.2715)	(2.1266)
	**[2.0770]**	**[0.7657]**	**[1.9622]**	**[1.3212]**	**[2.5613]**
Household head characteristics	Yes	Yes	Yes	Yes	Yes
Household variables	Yes	Yes	Yes	Yes	Yes
Year fixed effects	No	No	No	No	No
Regional fixed effects	Yes	Yes	Yes	Yes	Yes
**First-Stage**					
Distance to the nearest bank (km)	−0.0018***	−0.0018***	−0.0018***	−0.0018***	−0.0018***
	(0.0005)	(0.0005)	(0.0005)	(0.0005)	(0.0005)
F-statistic of first regression	12.36	12.36	12.36	12.36	12.36
Observations	16,742	16,742	16,742	16,742	16,742
***Panel C: GLSS7***					
Financial inclusion	2.1324*	−2.6457*	5.1060***	5.2124***	1.5606***
	(1.2896)	(1.5545)	(1.3309)	(1.7507)	(0.4198)
	**[0.3832]**	**[-0.4722]**	**[1.2969]**	**[0.8533]**	**[1.2303]**
Household head characteristics	Yes	Yes	Yes	Yes	Yes
Household variables	Yes	Yes	Yes	Yes	Yes
Year fixed effects	No	No	No	No	No
Regional fixed effects	Yes	Yes	Yes	Yes	Yes
**First-Stage**					
Distance to the nearest bank (km)	−0.0041***	−0.0041***	−0.0041***	−0.0041***	−0.0041***
	(0.0010)	(0.0010)	(0.0010)	(0.0010)	(0.0010)
F-statistic of first regression	18.22	18.22	18.22	18.22	18.22
Observations	6936	6936	6936	6936	6936

Robust standard errors in parentheses ***p < 0.01, **p < 0.05, *p < 0.1.

Standardised coefficients in square brackets.

Specifically, a rise in the financial inclusion's standard deviation in Column 1 of Panel A is linked to an increase in total residential energy expenditure by 1.2835 standard deviation. From Columns 2 to 5, a rise in the financial inclusion's standard deviation is linked with increases in residential energy expenditures by 0.3029, 1.3566, 1.1075, and 1.5158 standard deviations, respectively, for firewood/charcoal, LPG, electricity, and kerosene. Column 1 of Panel B shows that increasing financial inclusion's standard deviation by one is connected with an increase in total residential energy expenditure by 2.0770 standard deviation. In the same manner, we observe that a standard deviation increase in financial inclusion is associated with increases of 0.7657, 1.9622, 1.3212, and 2.5613 standard deviations, respectively, for firewood/charcoal, LPG, electricity and kerosene (in Column 2 to 5). The estimate in Column 1 of Panel C reveals that as financial inclusion's standard deviation increases by one, there is an increase in total residential energy expenditure by 0.3832. From Columns 3 to 5, the results show that a standard deviation increase in financial inclusion is associated with increases of 1.2969, 0.8533 and 1.2303 standard deviations in household expenses on LPG, electricity and kerosene, respectively. However, a standard deviation increase in financial inclusion is associated with a 0.4722 decrease in the residential energy expenditure on firewood/charcoal (Column 2). A potential reason for the negative coefficient for firewood/charcoal in the GLSS7 can be attributed to households' transition to environmentally friendly fuel choices due to an increased ability to pay emanating from enhanced financial inclusion.

### 5.1 Rural-urban analyses

To explore heterogeneities in our findings, we carried out disaggregated analyses on how financial inclusion influences residential energy expenditure for rural and urban households separately. Table 3, Panel A, Column 1 shows that increasing the standard deviation of financial inclusion by one is related to an increase in total residential energy expenditure by 0.8004 standard deviations in rural households. In Columns 2 to 5, a rise in the financial inclusion's standard deviation by one in rural households is linked with an increase in residential energy expenditure by 0.4903, 0.3302, 0.6136 and 0.6176 standard deviations, respectively, for firewood/charcoal, LPG, electricity and kerosene for rural households. As displayed in Column 1 of Panel B, an increase in financial inclusion for urban households by one standard deviation is connected to an increase of 2.6280 standard deviations in total residential energy expenditure. From Columns 3 to 5 of Panel B, a rise in the financial inclusion's standard deviation by one in urban households is associated with an increase in residential energy expenditure by 5.1929, 3.3154, and 1.8669 standard deviations, respectively, for LPG, electricity, and kerosene for urban households. However, a standard deviation increase in financial inclusion is associated with a 1.3857 standard deviation decrease in expenditure on firewood/charcoal. This implies that an increase in financial inclusion for urban households results in shifting preference from firewood/charcoal to other energy sources. An increase in access to financial services boosts households' income, which supports their ability to utilize clean or environmentally friendly fuels. The finding is in line with Adusah-Poku and Takeuchi [13] and Karimu, Mensah [11], who indicated that financially included individuals use clean energies.

**Table 3:** Financial inclusion and residential energy expenditure (2SLS regression):Rural-Urban.

VARIABLES	(1)	(2)	(3)	(4)	(5)
	Total	firewood	gas	electricity	kerosene
***Panel A: Pooled data for rural sample***					
Financial inclusion	4.6591***	2.4937***	0.7124***	3.3272***	1.6166***
	(0.7406)	(0.5795)	(0.1854)	(0.6106)	(0.3288)
	**[0.8004]**	**[0.4903]**	**[0.3302]**	**[0.6136]**	**[0.6176]**
Household head characteristics	Yes	Yes	Yes	Yes	Yes
Household variables	Yes	Yes	Yes	Yes	Yes
Year fixed effects	Yes	Yes	Yes	Yes	Yes
Regional fixed effects	Yes	Yes	Yes	Yes	Yes
**First-Stage**					
Distance to the nearest bank (km)	−0.0050***	−0.0050***	−0.0050***	−0.0050***	−0.0050***
	(0.0005)	(0.0005)	(0.0005)	(0.0005)	(0.0005)
F-statistic of first regression	91.08	91.08	91.08	91.08	91.08
Observations	13,280	13,280	13,280	13,280	13,280
***Panel A: Pooled data for rural sample***					
Financial inclusion	8.7598***	−7.0268***	4.5598***	3.7243***	4.1113***
	(2.6958)	(2.5127)	(1.3258)	(1.1076)	(1.3762)
	**[2.6280]**	**[-1.3857]**	**[5.1929]**	**[3.3154]**	**[1.8669]**
Household head characteristics	Yes	Yes	Yes	Yes	Yes
Household variables	Yes	Yes	Yes	Yes	Yes
Year fixed effects	Yes	Yes	Yes	Yes	Yes
Regional fixed effects	Yes	Yes	Yes	Yes	Yes
Distance to the nearest bank (km)	−0.0028***	−0.0028***	−0.0028***	−0.0028***	−0.0028***
	(0.0008)	(0.0008)	(0.0008)	(0.0008)	(0.0008)
F-statistic of first regression	11.48	11.48	11.48	11.48	11.48
Observations	10,398	10,398	10,398	10,398	10,398

Robust standard errors in parentheses ***p < 0.01, **p < 0.05, *p < 0.1. Standardised coefficients in square brackets.

Most urban residents live in self-contained or enclosed households, making using biomass fuel like charcoal inappropriate for them. Our finding is consistent with that of Adusah-Poku and Takeuchi [13], who found that rural households consume more firewood than urban households. Comparing the total residential energy expenditure estimates for the two locations, we can deduce that improvement in financial inclusion increases the ability to spend more on energy expenditure in urban areas. This result is because urban households’ access to financial services is greater than their rural counterparts in Ghana [69]. Overall, the urban and rural disaggregated analysis and the other analysis (baseline and 2SLS) are mainly in agreement.

### 5.2 Sensitivity/robustness checks

To test the robustness of the results generated, we engaged in several sensitivity checks in this subsection. First, we check the robustness of the standard 2SLS results in Table 2 using the Lewbel (2012) 2SLS method, which combines external and internally generated instruments and presents the results in Table 4. The report from the Lewbel 2SLS approach depicts a significant impact of financial inclusion on total energy expenditure. Generally, the positive financial inclusion and residential energy expenditure association results obtained from the standard 2SLS model are reinforced by the Lewbel 2SLS approach. For instance, in Panel A (Column 1), we establish that a rise in the financial inclusion's standard deviation by one is connected to an increase in the standard deviation of total residential energy expenditure by 0.0512. In Columns 3 and 4, a rise in the financial inclusion's standard deviation by one is linked with an increase in residential energy expenditure by 0.4945 and 0.1086 standard deviations, respectively, for LPG and electricity. The relationship between financial inclusion and household expenditure on kerosene was insigniﬁcant (see Column 5).

**Table 4:** Financial inclusion and residential energy expenditure (Lewbel 2SLS—Int & Ext).

VARIABLES	(1)	(2)	(3)	(4)	(5)
	Total Energy expenditure	Firewood/Charcoal	LPG	Electricity	Kerosene
***Panel A: Pooled data***					
Financial inclusion	0.2752**	−0.4013**	1.7787***	0.6022***	0.1107
	(0.1369)	(0.1731)	(0.1398)	(0.1503)	(0.0765)
	**[0.0512]**	**[-0.0767]**	**[0.4945]**	**[0.1086]**	[0.0464 [
Observations	23,678	23,678	23,678	23,678	23,678
R-squared	0.3507	0.1319	0.0872	0.3362	0.0351
***Panel B: GLSS6***					
Financial inclusion	0.0494	−0.3858**	1.6259***	0.2244*	−0.0852
	(0.1364)	(0.1802)	(0.1427)	(0.1339)	(0.0944)
	[0.0095]	**[-0.0769]**	**[0.4709]**	**[0.0429]**	[-0.0319]
Observations	16,742	16,742	16,742	16,742	16,742
R-squared	0.4043	0.1374	0.1356	0.4376	0.0178
***Panel C: GLSS7***					
Financial inclusion	0.5558**	−0.9650***	0.9818***	0.3562	0.1364*
	(0.2749)	(0.3446)	(0.2536)	(0.2934)	(0.0705)
	**[0.0999]**	**[-0.1722]**	**[0.2494]**	[0.0583]	**[0.1075]**
Observations	6936	6936	6936	6936	6936
R-squared	0.2552	0.0655	0.1774	0.2516	−0.0035

Robust standard errors in parentheses ***p < 0.01, **p < 0.05, *p < 0.1. Standardised coefficients in square brackets.

In Columns 3 and 4 of Panel B, a standard deviation increase in financial inclusion is connected with increases of 0.4709 and 0.0429 standard deviations in residential energy expenditure for LPG and electricity, respectively. The relationship between financial inclusion and residential energy expenditure was not significant in Columns 1 and 5. In Column 1 of Panel C, a standard deviation increase by one in financial inclusion is associated with an increase in total residential energy expenditure by 0.0999 standard deviation. The results in Columns 3 and 5 of Panel C depicted that a standard deviation increase in financial inclusion is linked with an increase in residential energy expenditure on LPG and electricity by 0.2494 and 0.1075 standard deviations, respectively. Again, the relationship between financial inclusion and energy expenditure on kerosene was insignificant (see Column 4).

However, in Column 2 of all the Panels, we observe that an increase in financial inclusion is associated with decreases in households’ expenditure on firewood/charcoal. Also, we observed that the positive nexus between financial inclusion and household LPG and electricity energy expenditure is more prominent. An explanation for this outcome is that financial inclusion enables residents to shift from dirty/solid fuel consumption to clean/modern fuels [16,20]. In sum, the results serve as evidence that our estimates are robust.

Second, we depend on the pooled data to assess the sensitivity or results to alternative weights for the four dimensions of the financial inclusion index. While a similar weight of 0.25 was initially assigned to each dimension in the previous analyses, our sensitivity analysis contains four separate models where larger weights are assigned to each dimension (see Table 5). The results of the financial inclusion index reported in Panel A are achieved by assigning a weight of 0.4 to “bank account”, while the three dimensions get 0.2 assignment each. In Panel B, the financial inclusion index is achieved by assigning a weight of 0.4 to “access to credit”, while the three dimensions get 0.2 assignments each. In Panel C, we assign a weight of 0.4 to “insurance”, while the other three dimensions get 0.2 assignments each. Similarly, in Panel D, we assign a weight of 0.4 to “receipt of financial remittances”, while the other three dimensions get 0.2 assignments each. After assigning relatively bigger weights to the different dimensions of the financial inclusion index, the results generated in Table 5 are mainly coherent with our findings above, conﬁrming that our estimates are robust to different weighting schemes.

**Table 5:** Financial inclusion and residential energy expenditure (Different weights of financial inclusion).

VARIABLES	(1)	(2)	(3)	(4)	(5)
	Total Energy expenditure	Firewood/Charcoal	LPG	Electricity	Kerosene
**Panel A: Weight of 0.4 for bank account**					
Financial inclusion	0.3232***	0.1966***	0.4039***	0.2299***	−0.0399*
	(0.0339)	(0.0436)	(0.0292)	(0.0347)	(0.0215)
	**[0.0598]**	**[0.0378]**	**[0.1128]**	**[0.0423]**	**[-0.0144]**
					
Observations	16,742	16,742	16,742	16,742	16,742
R-squared	0.4471	0.1622	0.3130	0.4812	0.1067
**Panel B: Weight of 0.4 for access to credit**					
Financial inclusion	0.2425***	0.1997***	0.2288***	0.1604***	−0.0030
	(0.0407)	(0.0527)	(0.0369)	(0.0420)	(0.0259)
	**[0.0343]**	**[0.0294]**	**[0.0489]**	**[0.0226]**	−0.0008
					
Observations	16,742	16,742	16,742	16,742	16,742
R-squared	0.4453	0.1618	0.3050	0.4803	0.1066
**Panel C: Weight of 0.4 for insurance**					
Financial inclusion	0.2773***	0.1306***	0.4412***	0.2008***	0.0046
	(0.0344)	(0.0448)	(0.0306)	(0.0353)	(0.0230)
	**[0.0489]**	**[0.0239]**	**[0.1174]**	**[0.0352]**	0.0016
					
Observations	16,742	16,742	16,742	16,742	16,742
R-squared	0.4462	0.1615	0.3143	0.4808	0.1066
**Panel D: Weight of 0.4 for remittance**					
Financial inclusion	0.2779***	0.2489***	0.1549***	0.1295***	0.0349
	(0.0362)	(0.0461)	(0.0304)	(0.0367)	(0.0237)
	**[0.0450]**	**[0.0419]**	**[0.0379]**	**[0.0209]**	0.0110
Observations	16,742	16,742	16,742	16,742	16,742
R-squared	0.4461	0.1627	0.3041	0.4802	0.1067

Robust standard errors in parentheses ***p < 0.01, **p < 0.05, *p < 0.1. Standardised coefficients in square brackets.

#### 5.2.1 Further subsampled analysis

Aside from robustness check and locational subgroup analysis, the association between financial inclusion and total residential energy expenditure across different wealth quintiles and household structures are also explored (see Table 6, Table 7) to add to the literature. Concerning the wealth quintiles (Table 6), the results reveal that a standard deviation increase in financial inclusion is respectively associated with increases of 0.0708, 0.0488, 0.0410, 0.0328, and 0.0490 standard deviations in residential energy expenditures for the poorest, poor, moderate, rich and richest household. Generally, our results suggest that the most beneficiaries in the financial inclusion-energy expenditure nexus are households in the lowest wealth quintile (i.e., the poorest). This provides evidence of a pro-poor policy available to policymakers who intend to improve the consumption of modern lighting and cooking fuels for households at the bottom of the pyramid.

**Table 6:** Financial inclusion and total residential energy expenditure (2SLS regression): Different wealth quintiles.

Variables	(1)	(2)	(3)	(4)	(5)
	Wealth quintiles				
	Poorest	Poor	Moderate	Rich	Richest
Financial inclusion	0.4554***	0.2765***	0.2066***	0.1474***	0.1839***
	(0.0852)	(0.0752)	(0.0652)	(0.0567)	(0.0445)
	**[0.0708]**	**[0.0488]**	**[0.0410]**	**[0.0328]**	**[0.0490]**
Household head characteristics	Yes	Yes	Yes	Yes	Yes
Household variables	Yes	Yes	Yes	Yes	Yes
Year fixed effects	Yes	Yes	Yes	Yes	Yes
Regional fixed effects	Yes	Yes	Yes	Yes	Yes
Observations	4899	4200	4149	4689	5741
R-squared	0.2594	0.3249	0.3353	0.3430	0.3017

Robust standard errors in parentheses ***p < 0.01, **p < 0.05, *p < 0.1 Standardised coefficients in square brackets.

**Table 7:** Financial inclusion and energy expenditure (2SLS results): household structures.

Variables	(1)	(2)	(3)	(4)
	Household structures			
	Female-headed single parent	Male-headed single parent	Female-headed dual parent	Male-headed dual parent
Financial inclusion	0.4361***	0.2408*	0.5548**	0.4148***
	(0.0972)	(0.1265)	(0.2567)	(0.0403)
	**[0.0922]**	**[0.0459]**	**[0.1120]**	**[0.0729]**
Household head characteristics	Yes	Yes	Yes	Yes
Household variables	Yes	Yes	Yes	Yes
Year fixed effects	Yes	Yes	Yes	Yes
Regional fixed effects	Yes	Yes	Yes	Yes
Observations	1716	1208	260	12,600
R-squared	0.3693	0.4027	0.4437	0.4486

Robust standard errors in parentheses ***p < 0.01, **p < 0.05, *p < 0.1 Standardised coefficients in square brackets.

In Table 7, the estimated results across the different household structures depict that a standard deviation increase in financial inclusion is linked to increases of 0.0922, 0.0459, 0.1120, and 0.0729 standard deviations in residential energy expenditures across a female-headed single parent, male-headed single parent, female-headed dual parent and male-headed dual-parent households, respectively. The most beneficiaries in the financial inclusion-energy expenditure nexus are female-headed dual-parent households, which could emanate from the resource-pooling effect of spouses living in the same household.

### 5.3 Potential channels

In this section, we use a two-step technique to investigate the potential role of household net income per capita. We explore the potential mediation role of income because entrepreneurship has already been used as a covariate in the baseline analysis. Koomson and Danquah [21] and Awaworyi Churchill and Smyth [72] have employed this method in their studies. The first step is to determine whether a significant link exists between financial inclusion and household income per capita. Table 8 shows that a standard deviation increase in financial inclusion is associated with a household net income per capita increase by 0.1427 standard deviation.

**Table 8:** Financial inclusion and household income.

Variables	(1)
	log (household net income)
Financial inclusion	0.4443***
	(0.0190)
	**[0.1427]**
Household head characteristics	Yes
Household variables	Yes
Year fixed effects	Yes
Regional fixed effects	Yes
Observations	21,915
R-squared	0.2650

Robust standard errors in parentheses ***p < 0.01 Standardised coefficients in square brackets.

In the next step, household net income per capita is introduced as a covariate in the energy expenditure model in Table 9. If net income per capita is a mediator, incorporating it will either reduce the magnitude of the previously obtained coefficient of financial inclusion or eliminate its significance. Table 9 (column 2) model is the same as the baseline model reported in Column 1 of Panel A in Table 1 but has been re-estimated using the same sample in Column 1 of Table 9. This is done to ensure that the estimates with and without the potential mediator are produced from the same sample and to provide a justifiable basis for comparison. Table 9 (column 1) shows that a one standard deviation increase in household net income per capita is linked to a 0.0684 standard deviation increase in residential energy expenditure. In Column 1, we ﬁnd that the magnitude of the coefficient of financial inclusion reduces after including the household net income per capita as a control variable in the residential energy expenditure model. The current analysis implies that household net income per capita is an important channel through which financial inclusion affects residential energy expenditure.

**Table 9:** Financial inclusion and residential energy expenditure (potential channel analysis).

Variables	(1)	(2)
	Total Energy expenditure	Total Energy expenditure
Financial inclusion	0.3149***	0.3671***
	(0.0296)	(0.0295)
	**[0.0589]**	**[0.0686]**
log (household net income)	0.1175***	
	(0.0107)	
	**[0.0684]**	
Household head characteristics	Yes	Yes
Household variables	Yes	Yes
Year fixed effects	Yes	Yes
Regional fixed effects	Yes	Yes
Observations	21,915	21,915
R-squared	0.4059	0.4025

Robust standard errors in parentheses ***p < 0.01 Standardised coefficients in square brackets.

## 6 Conclusion and policy implications

Despite the intervention by national governments and other non-governmental agencies to transition households to cleaner and modern fuel sources globally, the challenge in developing countries is still high. Although recent studies have pointed to the capability of financial inclusion in the energy transition process, literature on how it influences residential energy expenditure and its decomposed components remains scant. Also, existing studies linking financial inclusion to residential energy consumption have employed narrow measures rather than a multidimensional financial inclusion index. In this study, we address these gaps by employing two rounds of a comprehensive household survey in Ghana to examine how financial inclusion influences residential energy expenditures. The issue of endogeneity of financial inclusion is also addressed in this study. Our study also explored several heterogeneities in the financial inclusion-residential energy expenditure nexus.

In a nutshell, we observed that financial inclusion positively influences residential energy expenditure. The robustness of this result is confirmed through other sensitivity checks, including alternative weighting schemes for the financial inclusion index. We also found that financial inclusion increases residential energy expenditure more for urban households than their rural counterparts. Financial inclusion also enhances poor households' residential energy expenditure more than wealthy households. Our ﬁndings further revealed that households’ net income serves as an important channel through which financial inclusion transmits to residential energy expenditure.

Based on our results, it is important that national governments and policymakers in most developing nations, who are more likely to experience similar issues, prioritize financial inclusion policies to enhance households' transition to modern and cleaner fuels. For example, in order to encourage financial institutions to expand their branches, infrastructural incentives, including good roads and information and communication technology (ICT), should be provided in various communities, especially rural areas. When this happens, individuals will be willing to enjoy financial services due to reductions in transaction costs occurring from long distances, hence enhancing financial inclusion. Developing countries, particularly those in Africa, have adopted and are widely using mobile money technology. Policymakers can enhance this by increasing advocacy on its safe usage and ensuring that operators guarantee safety. The government can also induce the private sector to invest more in other financial technologies (fintech) since that enables more people to be financially included. Since this is technology-based, it will require some basic education for the section of the population unfamiliar with these technologies. Banks can also help in making account opening easier and faster for people. The conventional formalities surrounding traditional banking in Ghana, for example, make many poor and uneducated ones shun it. To enhance financial inclusion, the banks can make it a point of simplifying the accounts opening process, doing away with account opening fees and the provision of grantors in some cases. Banks can also go to the marketplaces where lots of people can be found to help them open accounts. This is actually something many banks are doing now in Ghana. Policymakers and regulatory bodies in the financial sector should also establish structures needed to reduce the average distance to banks since the long distance to banks is considered a factor that causes financial exclusion, an influencing factor of residential energy expenditure. Banking can be brought easily to people's doorsteps by the opening of many ATMs near marketplaces and residential areas. Banks can also provide training to their customers (to those who need it) to be able to access services online on their phones. One other suggestion that can enhance financial inclusion is the simplification of the acquisition of credit from financial institutions. Making a formal job provision of guarantors and collateral has been the main requirement for many financial institutions. These exclude a chunk of the population who are market folks, farmers and petty traders. With Ghana currently rolling out a national identification system and enhancing the addressing system, a more credible credit reference bureau can be established to reduce the chances of default. This will enhance many to be able to access credit in the financial market, hence enhancing financial inclusion.

Data limitations restricted our potential channel analysis to household net income, irrespective of the available avenues discussed in Section 2. Incoming studies can consider the other possible alternative avenues shown in the literature. Future studies could consider construcing a pseudo-panel dataset at the community or regional level using both the GLSS6 and GLSS7 to examine community or regional differences in Ghana.

## Data availability statement

All data to support our analysis and conclusion are publicly available on the Ghana Statistical Service Website.

https://www.statsghana.gov.gh/gssdatadownloadspage.ph.

## CRediT authorship contribution statement

**Martinson Ankrah Twumasi:** Writing – original draft, Visualization, Validation. **Frank Adusah-Poku:** Writing – original draft, Supervision, Conceptualization. **Alex O. Acheampong:** Writing – original draft, Visualization, Validation, Formal analysis. **Eric Evans Osei Opoku:** Writing – original draft, Visualization, Validation, Software, Methodology.

## Declaration of competing interest

The authors declare that they have no known competing financial interests or personal relationships that could have appeared to influence the work reported in this paper.

## Figures and Tables

**Table A1:** Summary statistics

Variable	Description	Mean	Std. Dev
Total energy expenditure	Continuous variable for total household energy expenditure	306.1827	639.9491
Firewood/Charcoal	Continuous variable for total household energy expenditure	92.9575	286.6368
Gas	Continuous variable for household expenditure on gas	24.3822	96.1465
Electricity	Continuous variable for household expenditure on electricity	182.7978	522.5463
Kerosene	Continuous variable for household expenditure on kerosene	6.0452	32.3199
Financial inclusion	Dummy variable equals 1 if household head is financially included	0.3504	0.4771
Female	Binary variable equals 1 if household head is female	0.2912	0.4543
Married	Binary variable equals 1 if household head is married	0.5784	0.4938
Educated	Binary variable equals 1 if household head is educated	0.4840	0.4998
Age	Age of the household head	45.9756	15.8972
Household size	Number of persons in the household	4.2430	2.8049
Rural	Binary variable equals 1 if household is in a rural area	0.5609	0.4963
Retired/inactive	Binary variable equals 1 if household head is retired/inactive	0.0889	0.2847
Employee	Binary variable equals 1 if household head is an employee	0.2180	0.4129
Self-employed	Binary variable equals 1 if household head is self-employed	0.6471	0.4779
Owns fridge or freezer	Binary variable equals 1 if household owns a fridge or freeze	0.2830	0.4505
Owns Radio or TV	Binary variable equals 1 if household owns a radio or TV	0.6651	0.4720
semi-detached/Flat/apartment	Binary variable equals 1 if household lives in semi-detached/Flat/apartment	0.0977	0.2969
Compound house	Binary variable equals 1 if household lives in a compound house	0.5555	0.4969
Huts/Buildings/Other	Binary variable equals 1 if household lives in a huts/buildings/other	0.1677	0.3736
Number of rooms in house	Number of rooms in household	2.2101	1.6722
Distance to the nearest bank	Average distance to the nearest bank measured in kilometres	14.3302	7.0670
Net income	Continuous variable for household's total net income	2162.7770	383,562.10

**Table A2:** Financial inclusion and residential energy expenditure (Baseline results)

Variables	(1)	(2)	(3)	(4)	(5)
	Total Energy expenditure	Firewood/Charcoal	Gas	Electricity	Kerosene
Financial inclusion	0.3765***	0.2961***	0.2994***	0.2377***	−0.0350**
	(0.0283)	(0.0346)	(0.0227)	(0.0306)	(0.0154)
	**[0.0700]**	**[0.0566]**	**[0.0832]**	**[0.0429]**	**[-0.0147]**
Female-headed household	0.4878***	0.7110***	0.1811***	0.1621***	0.1595***
	(0.0329)	(0.0395)	(0.0255)	(0.0353)	(0.0187)
	[0.0864]	[0.1293]	[0.0480]	[0.0278]	[0.0637]
married	0.1066***	0.3299***	0.2107***	0.0700**	0.0250
	(0.0318)	(0.0374)	(0.0236)	(0.0332)	(0.0183)
	[0.0205]	[0.0652]	[0.0606]	[0.0131]	[0.0109]
Educated	0.4826***	0.0492	0.3364***	0.6185***	−0.0792***
	(0.0315)	(0.0366)	(0.0215)	(0.0337)	(0.0173)
	[0.0940]	[0.0098]	[0.0980]	[0.1168]	[-0.0348]
Age of household head	−0.0025**	0.0022**	−0.0061***	−0.0050***	0.0032***
	(0.0010)	(0.0010)	(0.0006)	(0.0010)	(0.0005)
	[-0.0152]	[0.0143]	[-0.0561]	[-0.0298]	[0.0452]
Household size	0.0419***	0.1244***	−0.0346***	0.0011	0.0171***
	(0.0063)	(0.0070)	(0.0039)	(0.0063)	(0.0033)
	[0.0458]	[0.1397]	[-0.0565]	[0.0012]	[0.0422]
Rural	−1.5226***	−1.3381***	−0.3873***	−1.3060***	0.0566***
	(0.0309)	(0.0383)	(0.0238)	(0.0352)	(0.0175)
	[-0.2946]	[-0.2659]	[-0.1120]	[-0.2449]	[0.0247]
**Employment Status Base = Unemployed)**					
Retired/Inactive	0.0663	0.0084	0.1872***	0.0488	0.0722**
	(0.0816)	(0.0919)	(0.0538)	(0.0846)	(0.0356)
	[0.0074]	[0.0010]	[0.0311]	[0.0052]	[0.0181]
Employee	0.3090***	−0.0327	0.3572***	0.2520***	0.0528*
	(0.0714)	(0.0839)	(0.0525)	(0.0755)	(0.0293)
	[0.0497]	[-0.0054]	[0.0860]	[0.0393]	[0.0192]
Self Employed	−0.0732	−0.1091	0.0194	0.0024	0.0205
	(0.0686)	(0.0772)	(0.0441)	(0.0701)	(0.0266)
	[-0.0136]	[-0.0209]	[0.0054]	[0.0004]	[0.0086]
Owns fridge or freezer	1.0750***	0.2437***	1.0448***	1.4221***	−0.0608***
	(0.0302)	(0.0432)	(0.0328)	(0.0366)	(0.0178)
	[0.1888]	[0.0440]	[0.2743]	[0.2421]	[-0.0241]
Owns Radio or TV	−0.3688***	−0.1586***	0.0050	−0.4464***	0.0487***
	(0.0287)	(0.0341)	(0.0218)	(0.0310)	(0.0141)
	[-0.0679]	[-0.0300]	[0.0014]	[-0.0796]	[0.0202]
**Dwelling Type (Base = Separate house)**					
semi-detached/Flat/apartment	0.0294	−0.1943***	0.1700***	0.0489	0.0176
	(0.0526)	(0.0617)	(0.0452)	(0.0588)	(0.0272)
	[0.0034]	[-0.0231]	[0.0294]	[0.0055]	[0.0046]
Compound house	0.2444***	0.2043***	−0.1377***	0.3691***	0.0334*
	(0.0379)	(0.0438)	(0.0286)	(0.0411)	(0.0193)
	[0.0473]	[0.0406]	[-0.0399]	[0.0693]	[0.0146]
Huts/Buildings/Other	−0.3974***	−0.1156**	−0.1284***	−0.4728***	0.1235***
	(0.0514)	(0.0528)	(0.0294)	(0.0501)	(0.0265)
	[-0.0579]	[-0.0173]	[-0.0280]	[-0.0667]	[0.0406]
Number of rooms in house	0.0555***	−0.0864***	0.1027***	0.0756***	−0.0041
	(0.0104)	(0.0119)	(0.0080)	(0.0106)	(0.0058)
	[0.0362]	[-0.0578]	[0.1001]	[0.0478]	[-0.0060]
Year fixed effect	Yes	Yes	Yes	Yes	Yes
Regional fixed effect	Yes	Yes	Yes	Yes	Yes
Constant	3.9218***	1.7492***	0.4660***	3.1027***	−0.0289
	(0.1028)	(0.1169)	(0.0711)	(0.1087)	(0.0455)
Observations	23,678	23,678	23,678	23,678	23,678
R-squared	0.4031	0.1646	0.2982	0.3949	0.1092

Robust standard errors in parentheses ***p < 0.01, **p < 0.05, *p < 0.1.

Standardised coefficients in square bracket.

## Footnotes

1 These regions are the Upper West, Northern, Upper East, Brong Ahafo and the Volta regions.

2 Details on the NFIDS can be found here. https://mofep.gov.gh/sites/default/files/acts/NFIDs_Report.pdf.

3 Energy poverty is sometimes linked to energy expenditure. In the UK and some European countries, energy poverty is defined using the share of energy expenditure in total household expenditure. Since there are no studies on financial inclusion and energy expenditure, we provide a review on the link between financial inclusion and energy poverty.
